# Death Anxiety in the Association Between Exposure-Related Burden of Patient Death and Occupational Burnout Among Healthcare Professionals: A Cross-Sectional Study

**DOI:** 10.3390/healthcare14152382

**Published:** 2026-08-04

**Authors:** Magdalena Mikulska, Małgorzata Treppner, Iwona Sadowska-Krawczenko, Aldona Katarzyna Jankowska

**Affiliations:** 1 Center for Clinical Communication, Collegium Medicum in Bydgoszcz, Nicolaus Copernicus University in Toruń, 85-077 Bydgoszcz, Poland; 2 Department of Medical Communication and Medical Humanities, Collegium Medicum in Bydgoszcz, Nicolaus Copernicus University in Toruń, 85-077 Bydgoszcz, Poland; k.jankowska@cm.umk.pl; 3 Institute of Psychology, Faculty of Social Sciences, University of Gdańsk, 80-309 Gdańsk, Poland; m.treppner.196@studms.ug.edu.pl; 4 Doctoral School at the Faculty of Social Sciences, University of Gdańsk, 80-309 Gdańsk, Poland; 5 Department of Neonatology, Collegium Medicum in Bydgoszcz, Nicolaus Copernicus University in Toruń, 85-077 Bydgoszcz, Poland; iwonasadowska@cm.umk.pl; 6 Polish Academy of Sciences Scientific Centre in Paris, 75116 Paris, France

**Keywords:** occupational burnout, healthcare workers, patient death, death anxiety, occupational stress, coping strategies, emotional burden

## Abstract

**Highlights:**

**What are the main findings?**

- Exposure-related burden of patient death was associated with occupational burnout among healthcare professionals.
- Death anxiety was involved in an exploratory indirect statistical association between exposure-related burden of patient death and occupational burnout.

**What are the implications of the main findings?**

- Death-related emotional and existential burden should be considered in burnout prevention.
- Healthcare organizations may benefit from organizational, administrative, and psychosocial support measures addressing death-related occupational burden.

**Abstract:**

Background/Objectives: Occupational burnout remains a major concern among healthcare professionals and is closely linked to chronic workplace stress. Exposure to patient death may represent a death-related psychosocial occupational burden that is emotionally and existentially demanding but still insufficiently examined in relation to burnout. This study aimed to examine associations between exposure-related burden of patient death, death anxiety, and occupational burnout among healthcare professionals, and to explore whether the observed pattern of results was consistent with an indirect statistical association involving death anxiety. Associations between coping strategies, burnout, and death anxiety were also examined. Methods: The study included 60 healthcare professionals representing various healthcare occupations. Data were collected using an anonymous online survey comprising a preliminary author-developed measure of exposure-related burden of patient death, a brief preliminary author-developed measure of death anxiety, the Burnout Assessment Tool (BAT-PL), and the Brief COPE inventory. Statistical analyses included descriptive statistics, Pearson correlation analyses, linear regression, and exploratory indirect effect analysis using Hayes’ PROCESS macro. Results: In the sample of 60 healthcare professionals, exposure-related burden of patient death was positively associated with occupational burnout (r = 0.53, 95% CI [0.32, 0.69], *p* < 0.001) and death anxiety (r = 0.30, 95% CI [0.05, 0.51], *p* = 0.022). Death anxiety was positively associated with occupational burnout (r = 0.54, 95% CI [0.33, 0.70], *p* < 0.001). The exploratory indirect effect analysis yielded a pattern of results consistent with an indirect statistical association involving death anxiety (B = 0.12, 95% CI [0.0001, 0.282]). The overall model accounted for 44.6% of the variance in occupational burnout (R^2^ = 0.446). Avoidant coping strategies were associated with higher levels of death anxiety and burnout, whereas emotion-focused coping strategies were associated with lower levels of death anxiety. Conclusions: The findings suggest that death-related emotional and existential burden may be relevant to understanding occupational burnout among healthcare professionals. Death anxiety may be one psychological factor involved in this association. However, because of the cross-sectional design, small convenience sample, and use of preliminary author-developed measures, the results should be interpreted cautiously and should not be considered evidence of causal mechanisms. These findings highlight the need to consider death-related experiences in occupational burnout prevention and worker well-being strategies in healthcare settings.

## 1. Introduction

Occupational burnout is a significant public health concern among healthcare professionals. Defined as a syndrome resulting from chronic work-related stress, burnout is characterized by exhaustion, mental distancing from work, and reduced cognitive and emotional functioning [1,2]. Numerous studies have reported a high prevalence of burnout among healthcare workers, with consequences extending beyond individual well-being to include reduced quality of patient care and adverse organizational outcomes [2,3]. In recent years, burnout levels among healthcare professionals have remained consistently high worldwide, further exacerbated by the COVID-19 pandemic and ongoing organizational challenges within healthcare systems [4,5].

From an occupational health perspective, burnout among healthcare professionals should be understood not only as an individual psychological outcome, but also as a worker well-being problem shaped by work organization, psychosocial hazards, and system-level resources [6,7]. This perspective is particularly relevant in medical professions, as systematic reviews have shown that burnout among physicians is a widespread concern, although prevalence estimates vary substantially depending on definitions and measurement methods [8]. Contemporary occupational health frameworks, including the NIOSH worker well-being framework and the Total Worker Health approach, emphasize the integration of hazard control with strategies aimed at promoting workers’ safety, health, and well-being [6,9].

One distinctive yet relatively understudied occupational burden in healthcare is exposure to patient death, which may be conceptualized as a death-related psychosocial occupational hazard.

In many medical specialties, encounters with dying patients constitute a routine aspect of clinical practice rather than an occasional event. Repeated exposure to death may represent a unique occupational stressor that extends beyond typical job demands, affecting not only professional functioning but also fundamental emotional and existential dimensions of human experience [10,11,12]. Previous studies conducted among healthcare professionals, including obstetrics and gynecology staff, suggest that experiences related to patient death may be associated with increased psychological burden, higher levels of occupational burnout, and more negative perceptions of death [13].

One psychological factor that may be involved in the association between exposure-related burden of patient death and occupational burnout is death anxiety. Encounters with dying patients may trigger reflections on one’s own mortality and intensify existential concerns, a relationship that can be understood within the framework of Terror Management Theory [14,15]. Recent evidence indicates that death anxiety among healthcare professionals is associated with a range of psychological and occupational factors, including job stress, burnout, avoidance of dying patients, mental health status, coping with death, and perceived self-efficacy [16]. This supports the consideration of death anxiety as a death-specific psychological factor that is conceptually distinct from broader constructs such as secondary traumatic stress or compassion fatigue [17].

Another factor that may influence responses to death-related occupational stress is coping style. Different coping strategies, including problem-focused, emotion-focused, and avoidant coping, may function either as protective resources or as risk factors for psychological well-being among healthcare professionals [18]. Therefore, coping strategies were included as an exploratory component of the present study.

Despite the growing body of literature on occupational burnout among healthcare professionals, limited research has examined death anxiety as a specific psychological factor involved in death-related occupational stress. Previous studies have focused primarily on general occupational stress, secondary traumatic stress, compassion fatigue, or attitudes toward death. Although these constructs are highly relevant, they do not fully capture the existential dimension of repeated encounters with patient dying and death. Although our previous research identified an association between experiences related to patient death and occupational burnout among obstetrics and gynecology clinicians [13], it did not examine death anxiety as a psychological factor potentially involved in this relationship. The present study extends this line of research by examining death anxiety in the association between exposure-related burden of patient death and occupational burnout in a heterogeneous sample of healthcare professionals.

Accordingly, the primary aim of the present study was to examine associations between exposure-related burden of patient death, death anxiety, and occupational burnout, and to explore whether the observed pattern of results was consistent with an exploratory indirect statistical association involving death anxiety. A secondary, exploratory aim was to examine associations between coping strategies, occupational burnout, and death anxiety.

The following hypotheses were formulated:

**H1.**  *Exposure-related burden of patient death is positively associated with occupational burnout.*

**H2.**  *Exposure-related burden of patient death is positively associated with death anxiety.*

**H3.**  *Death anxiety is positively associated with occupational burnout.*

**H4.**  *The pattern of associations between exposure-related burden of patient death, death anxiety, and occupational burnout is consistent with an exploratory indirect statistical association involving death anxiety.*

## 2. Materials and Methods

### 2.1. Research Design

This study used a cross-sectional survey design. The aim was to examine associations between exposure-related burden of patient death, death anxiety, occupational burnout, and coping strategies among healthcare professionals. Because of the cross-sectional design, the study was intended to identify statistical associations rather than causal relationships or temporal mechanisms.

### 2.2. Study Population and Sample

The study included 60 healthcare professionals representing medical and allied health professions. Participants were eligible to take part if they were adults working in healthcare and had professional contact with patients. No restrictions were imposed regarding specific profession, workplace setting, or length of professional experience. The sample was recruited using convenience sampling through professional networks and social media.

The sample size was determined by the exploratory nature of the study and by the availability of healthcare professionals willing to participate in an anonymous survey on a sensitive occupational topic. No a priori power analysis was conducted. Therefore, the study should be regarded as an exploratory investigation aimed at identifying preliminary associations rather than providing population-level estimates. Given the final sample size of *N* = 60, the study was primarily suited to detecting medium-to-large associations, whereas small effects and more complex models may have remained underpowered.

### 2.3. Data Collection Method

Data were collected between August 2025 and February 2026 using an anonymous online questionnaire created and distributed via Google Forms. The questionnaire was distributed through the authors’ professional networks and social media channels. Participation was voluntary, and participants completed the questionnaire independently.

The questionnaire referred to participants’ professional experiences related to patient death. No fixed recall period was imposed; therefore, participants could refer to their broader professional experience when responding to the survey items. This should be considered when interpreting the findings, as the reported experiences may reflect different time frames across participants.

### 2.4. Measurement Instruments

The questionnaire included demographic and professional questions, a preliminary author-developed measure of exposure-related burden of patient death, a brief preliminary author-developed measure of death anxiety, the Burnout Assessment Tool (BAT-PL), and the Brief COPE inventory. For scale-based measures, total scores were calculated as mean item scores according to the response range of each instrument, with higher scores indicating higher levels of the measured construct or greater use of a given coping strategy.

#### 2.4.1. Exposure-Related Burden of Patient Death

Exposure-related burden of patient death was assessed using a preliminary author-developed seven-item measure designed for the purposes of this exploratory study. The items referred to professional experiences related to patient death, including both exposure to such events and the subjective emotional burden associated with them. No fixed recall period was imposed. Responses were rated on a five-point Likert scale ranging from 1 to 5. Two items were reverse-coded. The total score was calculated as a mean item score, with higher scores indicating greater exposure-related burden. Possible scores ranged from 1 to 5. The full item content is provided in Supplementary File S1.

#### 2.4.2. Death Anxiety

Death anxiety was assessed using a brief preliminary author-developed nine-item measure based on theoretical concepts of death anxiety and existing instruments, particularly the Death Anxiety subscale of the Death Anxiety and Fascination Scale developed by Żemojtel-Piotrowska and Piotrowski [19]. The items referred to emotional and cognitive reactions related to one’s own death, the death of significant others, and patient death. Responses were rated on a five-point Likert scale ranging from 1 to 5. The total score was calculated as a mean item score, with higher scores indicating higher death anxiety. Possible scores ranged from 1 to 5. The full item content is provided in Supplementary File S1.

#### 2.4.3. Occupational Burnout

Occupational burnout was assessed using the Polish adaptation of the Burnout Assessment Tool (BAT-PL), a 23-item instrument measuring exhaustion, mental distance, and impaired cognitive and emotional functioning [20]. Responses were rated on a five-point scale ranging from 1 to 5. The total burnout score was calculated as a mean item score, with higher scores indicating higher occupational burnout. Possible scores ranged from 1 to 5.

#### 2.4.4. Coping Strategies

Coping strategies were assessed using the Brief COPE inventory, a 28-item measure of coping responses to stressful situations [18]. Responses were rated on a four-point scale ranging from 0 to 3. In the present study, analyses included both individual coping strategies and three broader coping dimensions: problem-focused coping, emotion-focused coping, and avoidant coping. Scores were calculated as mean item scores, with higher scores indicating greater use of a given coping strategy or coping dimension. Possible scores ranged from 0 to 3.

### 2.5. Data Analysis

Statistical analyses were performed using IBM SPSS Statistics, version 29.0.2.0 (IBM Corp., Armonk, NY, USA). Descriptive statistics were calculated for demographic, professional, and study variables. Internal consistency of multi-item measures was assessed using Cronbach’s alpha coefficients. All submitted questionnaires included complete data for the variables analyzed in the present study; therefore, no imputation of missing data was performed.

The normality of distributions was evaluated using skewness and kurtosis values. As no substantial deviations from normality were observed, parametric statistical procedures were applied.

Associations between variables were examined using Pearson correlation coefficients. Where applicable, 95% confidence intervals were reported to facilitate interpretation of the strength and precision of the observed associations. Linear regression analysis was conducted to examine the association between exposure-related burden of patient death and occupational burnout.

An exploratory indirect effect analysis was conducted using Hayes’ PROCESS macro for SPSS, Model 4, with 5000 bootstrap samples. The analysis examined whether the pattern of associations between exposure-related burden of patient death, death anxiety, and occupational burnout was consistent with an indirect statistical association involving death anxiety. The indirect association was evaluated using 95% bootstrap confidence intervals. Statistical significance was set at *p* < 0.05.

### 2.6. Ethical Considerations

The study was conducted in accordance with the Declaration of Helsinki and was approved by the Faculty Committee for Ethics and Scientific Research of Nicolaus Copernicus University in Toruń (approval no. KEWL 7/2026; approval date: 30 July 2025).

Before completing the questionnaire, participants received information about the purpose of the study, the voluntary nature of participation, anonymity, data use, and the possibility to discontinue participation at any time without providing a reason. Electronic informed consent was obtained before participants proceeded to the questionnaire.

Participation in the study was voluntary, and no remuneration was offered. No identifying personal data were collected. The data were stored in a password-protected electronic file and were accessible only to the principal investigator and the member of the research team responsible for statistical analysis. Data were analyzed and reported in aggregated form.

## 3. Results

### 3.1. Sample Characteristics

The study included 60 healthcare professionals representing a range of medical and allied health professions. Detailed characteristics of the study sample are presented in Table 1.

Women accounted for 73.3% of participants, and the mean length of professional experience was 16.95 years (SD = 11.24). More than 80% of respondents reported encountering patient death at least occasionally, with 21.7% indicating very frequent exposure. Detailed information regarding professional background, age distribution, and workplace setting is presented in Table 1.

### 3.2. Reliability and Descriptive Statistics

Descriptive statistics and Cronbach’s alpha coefficients for the main study variables are presented in Table 2. Cronbach’s alpha values were 0.72 for the preliminary author-developed measure of exposure-related burden of patient death, 0.80 for the brief preliminary author-developed death anxiety measure, and 0.95 for the BAT-PL occupational burnout scale. The mean score for exposure-related burden of patient death was 3.33 (SD = 0.82), the mean death anxiety score was 2.78 (SD = 0.85), and the mean occupational burnout score was 2.37 (SD = 0.79). Possible and observed score ranges are presented in Table 2.

### 3.3. Associations Between Exposure-Related Burden of Patient Death, Death Anxiety, and Occupational Burnout

Pearson correlation analyses were conducted to examine associations between exposure-related burden of patient death, death anxiety, and occupational burnout. Correlation coefficients with 95% confidence intervals are presented in Table 3.

Exposure-related burden of patient death was positively associated with occupational burnout (r = 0.53, 95% CI [0.32, 0.69], *p* < 0.001) and death anxiety (r = 0.30, 95% CI [0.05, 0.51], *p* = 0.022). Death anxiety was positively associated with occupational burnout (r = 0.54, 95% CI [0.33, 0.70], *p* < 0.001).

### 3.4. Association Between Exposure-Related Burden of Patient Death and Occupational Burnout

A linear regression analysis was conducted to examine the association between exposure-related burden of patient death and occupational burnout.

The regression model was statistically significant (F = 22.71, *p* < 0.001) and accounted for 28.1% of the variance in occupational burnout (R^2^ = 0.281). Exposure-related burden of patient death was positively associated with occupational burnout (β = 0.53, t = 4.77, *p* < 0.001) (Table 4).

### 3.5. Exploratory Indirect Statistical Association Involving Death Anxiety

An exploratory indirect effect analysis using Hayes’ PROCESS macro (Model 4; 5000 bootstrap samples) was conducted to examine whether the pattern of associations between exposure-related burden of patient death, death anxiety, and occupational burnout was consistent with an indirect statistical association involving death anxiety.

Exposure-related burden of patient death was positively associated with death anxiety (B = 0.30, SE = 0.13, *p* = 0.022). Death anxiety was positively associated with occupational burnout after controlling for exposure-related burden of patient death (B = 0.39, SE = 0.10, *p* < 0.001).

The direct association between exposure-related burden of patient death and occupational burnout remained statistically significant after death anxiety was included in the model (B = 0.39, SE = 0.10, *p* < 0.001).

The indirect association involving death anxiety was statistically significant (B = 0.12, 95% CI [0.0001, 0.282]) (Table 5). The overall model accounted for 44.6% of the variance in occupational burnout (R^2^ = 0.446). Given the cross-sectional design, this result should be interpreted as an exploratory indirect statistical association rather than evidence of a causal mediating mechanism.

### 3.6. Coping Strategies: Exploratory Analyses

Among the coping strategies examined, acceptance (M = 2.28), active coping (M = 2.17), and planning (M = 2.10) showed the highest mean scores.

Avoidant coping strategies were positively associated with both occupational burnout (r = 0.51, 95% CI [0.29, 0.68], *p* < 0.001) and death anxiety (r = 0.37, 95% CI [0.13, 0.57], *p* = 0.004).

Emotion-focused coping strategies were negatively associated with death anxiety (r = −0.31, 95% CI [−0.52, −0.06], *p* = 0.018). A negative association between problem-focused coping and occupational burnout was observed, but it did not reach statistical significance (r = −0.25, 95% CI [−0.47, 0.00], *p* = 0.053).

## 4. Discussion

The aim of this study was to examine associations between exposure-related burden of patient death, death anxiety, and occupational burnout among healthcare professionals, and to explore whether the observed pattern of results was consistent with an indirect statistical association involving death anxiety. The findings indicate that exposure-related burden of patient death was positively associated with occupational burnout and death anxiety, and that death anxiety was positively associated with occupational burnout. The exploratory indirect effect analysis suggested an indirect statistical association involving death anxiety; however, given the cross-sectional design, this finding should not be interpreted as evidence of a causal psychological mechanism.

The positive association between exposure-related burden of patient death and occupational burnout is consistent with previous findings among obstetrics and gynecology staff, where experiences related to patient death were associated with higher burnout levels and more negative perceptions of death [13]. The present findings also align with research suggesting that exposure to potentially traumatic or emotionally demanding events in healthcare settings may be associated with secondary traumatic stress and occupational burnout [12,21]. However, the present study adds a death-specific perspective by focusing on subjective burden related to patient death and death anxiety, rather than on general occupational stress alone.

Exposure-related burden of patient death was also positively associated with death anxiety. This finding is consistent with the assumption that repeated encounters with dying patients may activate personal reflections on mortality, human vulnerability, and the limits of medical intervention. Such experiences may be understood in light of Terror Management Theory, which proposes that reminders of mortality can intensify existential concerns [14,15]. Recent evidence also suggests that death anxiety among healthcare professionals is associated with occupational and psychological factors such as job stress, burnout, avoidance of dying patients, mental health status, coping with death, and perceived self-efficacy [16].

The observed indirect statistical association suggests that death anxiety may be one psychological factor involved in the relationship between exposure-related burden of patient death and occupational burnout. This interpretation should remain cautious. The data do not allow conclusions about temporal order or causality. It is possible that repeated death-related burden contributes to higher death anxiety and burnout, but it is also possible that professionals experiencing higher burnout or distress perceive death-related experiences as more burdensome. Longitudinal studies are needed to clarify the direction of these associations.

From this perspective, patient death may be considered not only as a difficult clinical event, but also as a death-related psychosocial occupational burden. The present findings suggest that subjective burden related to patient death may be associated with both existential concerns and occupational burnout. This interpretation is consistent with theoretical models emphasizing the balance between job demands and available resources within the work environment [22]. However, the present study cannot determine whether death-related burden contributes to burnout over time, whether burnout increases the perceived burden of death-related experiences, or whether both are influenced by other individual and organizational factors.

The exploratory analyses of coping strategies showed that avoidant coping was associated with higher levels of both death anxiety and occupational burnout, whereas emotion-focused coping was associated with lower death anxiety. These findings are consistent with previous research linking avoidance-oriented coping with poorer psychological outcomes and support the need to consider coping styles in relation to death-related occupational burden. However, because these analyses were exploratory and based on cross-sectional data, they should not be interpreted as evidence that specific coping strategies directly increase or reduce burnout.

No statistically significant moderation effect of coping strategies was observed in the present sample. Given the relatively small sample size, this finding should be interpreted cautiously. The absence of statistically significant moderation does not exclude the possibility that coping strategies or organizational resources shape how healthcare professionals respond to patient death. Future studies with larger samples should examine whether individual coping resources and workplace-level supports modify the association between death-related burden, death anxiety, and occupational burnout.

### 4.1. Practical Implications

The findings of the present study may have implications for healthcare organizations and for initiatives aimed at supporting healthcare professionals who are repeatedly exposed to patient dying and death. In particular, they highlight the importance of considering the emotional and existential aspects of clinical work as part of occupational burnout prevention and worker well-being strategies.

From an occupational health perspective, exposure-related burden of patient death should not be addressed solely through individual coping strategies. In line with the hierarchy of controls applied to the NIOSH Total Worker Health approach, preventive actions should include organizational, administrative, environmental, and psychosocial levels of support [9]. Because patient death cannot be eliminated from many healthcare settings, interventions should focus on reducing cumulative emotional burden, strengthening system-level resources, and improving access to support after difficult death-related events.

At the environmental or engineering level, healthcare organizations may consider creating appropriate spaces and conditions for sensitive conversations, team reflection, and post-event support. At the administrative level, possible actions include structured procedures following particularly difficult patient deaths, regular supervision, workload monitoring, and clear referral pathways for psychological support. At the level of psychosocial support, which may function as a PPE-equivalent resource in the context of psychosocial occupational risk, interventions may include peer support, reflective discussion groups, structured debriefing, and access to psychological consultation. Training-based interventions, including simulation-based learning focused on communication about dying and death, may further help healthcare professionals prepare for the emotional, communicative, and existential dimensions of patient death [23].

These recommendations should not be understood as placing responsibility for burnout prevention solely on individual healthcare professionals. Individual coping strategies may be important complementary resources, but the findings of the present study support the need for organizational and system-level approaches to death-related occupational burden. Although the results should be interpreted cautiously, they suggest that interventions addressing experiences related to patient death and death anxiety may represent a valuable component of occupational burnout prevention programs in healthcare settings.

### 4.2. Limitations

Several limitations of the present study should be acknowledged. First, the study used a cross-sectional design, which precludes conclusions about causality or temporal order. Although the observed pattern of results was consistent with an indirect statistical association involving death anxiety, it cannot establish whether exposure-related burden of patient death contributes to death anxiety and burnout over time, whether burnout increases the perceived burden of death-related experiences, or whether these associations are influenced by other factors.

Second, the study was based on a relatively small convenience sample (*N* = 60), which limits statistical power, the stability of estimates, and the generalizability of the findings. No a priori power analysis was conducted. Therefore, the findings should be interpreted as preliminary and exploratory. The sample size was primarily suited to detecting medium-to-large associations, whereas small effects and more complex models may have remained underpowered. Future studies should use larger, multicenter samples and conduct an a priori power analysis to ensure adequate statistical power.

Third, the composition of the sample should be considered when interpreting the findings. Women constituted the majority of participants, and physicians were the largest professional group, whereas other healthcare professions, including nurses, midwives, paramedics, psychologists, and healthcare assistants, were represented by smaller numbers. This limits the possibility of drawing conclusions about specific professional groups or comparing occupational settings. Future studies should use more balanced and stratified samples to examine whether the associations observed in the present study differ across professions and healthcare contexts.

Fourth, the use of preliminary author-developed measures to assess exposure-related burden of patient death and death anxiety is an important limitation. Although both measures demonstrated acceptable internal consistency in the present sample, internal consistency alone is not sufficient to establish the validity of an instrument. Further studies are needed to evaluate their psychometric properties, including factor structure, convergent and discriminant validity, test–retest reliability, and criterion-related validity. Therefore, findings based on these measures should be interpreted cautiously and considered preliminary.

Fifth, the measure of patient death exposure included items referring both to exposure to patient death and to the subjective emotional burden associated with such exposure. For this reason, the measure should be interpreted as reflecting exposure-related burden rather than objective exposure alone. From an occupational health perspective, this distinction is important because the occupational hazard itself and the individual psychological response to that hazard represent different aspects of risk. Future studies should develop and validate separate measures of objective exposure to patient death, such as frequency, recency, type of death, degree of professional involvement, and participation in communication with the patient’s family, and subjective burden related to such exposure, such as emotional distress, helplessness, guilt, intrusive thoughts, and perceived need for support.

Sixth, no fixed recall period was imposed in the questionnaire. Participants could therefore refer to broader professional experiences related to patient death, which may have differed in recency and duration across respondents. This may have introduced recall bias and limits the precision of exposure assessment. Future studies should define specific time frames for exposure assessment and distinguish recent from cumulative professional experiences.

Seventh, the study relied on self-report data collected through an online questionnaire. As a result, the findings may be affected by self-report bias, common method bias, and self-selection bias. Healthcare professionals who had particularly strong experiences related to patient death or who were more interested in the topic may have been more likely to participate. In addition, because all variables were assessed using the same data collection method, associations between variables may have been inflated by shared method variance.

Finally, the analyses did not adjust for potential confounding variables, such as workload, workplace setting, profession, years of experience, organizational support, previous training in end-of-life communication, or personal experiences of loss. The study also did not collect occupational health indicators such as death-related adverse events, near-misses, formal risk assessment data, or organizational reporting procedures. Future research should incorporate these variables to better distinguish individual psychological responses from organizational and occupational risk factors.

## 5. Conclusions

The findings of this exploratory cross-sectional study indicate that exposure-related burden of patient death was positively associated with occupational burnout among healthcare professionals. Death anxiety was also positively associated with both exposure-related burden of patient death and occupational burnout, and the observed pattern of results was consistent with an exploratory indirect statistical association involving death anxiety.

These findings suggest that death-related emotional and existential burden may be relevant to understanding occupational burnout in healthcare settings. However, because of the cross-sectional design, small convenience sample, and use of preliminary author-developed measures, the results should be interpreted cautiously and should not be considered evidence of causal mechanisms.

Future studies should use larger, multicenter and longitudinal designs, validated instruments, and more precise measures distinguishing objective exposure to patient death from subjective burden related to such exposure. Further research should also examine organizational and occupational factors that may contribute to or protect against death-related burden and burnout among healthcare professionals.

## Figures and Tables

**Table 1:** Characteristics of the Study Sample (*N* = 60).

Variable	*n*	%
Gender		
Female	44	73.3
Male	16	26.7
Age group (years)		
26–35	17	28.3
36–45	17	28.3
46–55	16	26.7
56–65	10	16.7
Profession		
Physician	34	56.7
Nurse	9	15
Healthcare assistant	5	8.3
Paramedic	5	8.3
Midwife	3	5
Psychologist	2	3.3
Other	2	3.3
Workplace setting		
Hospital ward	23	38.3
Hospice	14	23.3
Emergency department	7	11.7
Emergency medical services	5	8.3
Other healthcare settings	11	18.4
Frequency of exposure to patient death		
Very often	13	21.7
Often	18	30
Sometimes	17	28.3
Rarely	10	16.7
Never	2	3.3

**Table 2:** Descriptive Statistics and Reliability Coefficients.

Variable	Possible Range	Observed Range	M	SD	Cronbach’s α
Exposure-related burden of patient death	1–5	1.71–4.86	3.33	0.82	0.72
Death anxiety	1–5	1.00–4.89	2.78	0.85	0.80
Occupational burnout	1–5	1.09–4.91	2.37	0.79	0.95

Skewness and kurtosis values did not indicate substantial deviations from normality, supporting the use of parametric statistical analyses.

**Table 3:** Correlations between Study Variables.

Variable	1	2	3
1. Exposure-related burden of patient death	—		
2. Death anxiety	0.30 * [0.05, 0.51]	—	
3. Occupational burnout	0.53 *** [0.32, 0.69]	0.54 *** [0.33, 0.70]	—

Note. Values are Pearson correlation coefficients with 95% confidence intervals in brackets. * *p* < 0.05; *** *p* < 0.001.

**Table 4:** Linear Regression Model for the Association between Exposure-Related Burden of Patient Death and Occupational Burnout.

**Predictor**	**β**	**t**	***p***
Exposure-related burden of patient death	0.53	4.77	<0.001
**R^2^**	**F**	**df**	***p***
0.281	22.71	1.58	<0.001

**Table 5:** Exploratory Indirect Statistical Association between Exposure-Related Burden of Patient Death and Occupational Burnout Involving Death Anxiety.

**Path**	**B**	**SE**	***p***
Exposure-related burden of patient death → Death anxiety	0.3	0.13	0.022
Death anxiety → occupational burnout	0.39	0.1	<0.001
Direct association: exposure-related burden of patient death → occupational burnout)	0.39	0.1	<0.001
**Effect**	**B**	**95% CI**	
Indirect association involving death anxiety	0.12	[0.0001, 0.282]	

Note. The exploratory indirect effect analysis was conducted using Hayes’ PROCESS macro (Model 4) with 5000 bootstrap samples. Given the cross-sectional design, the results should be interpreted as an indirect statistical association rather than evidence of a causal mediating mechanism. The arrow (→) indicates the direction of the tested statistical path and does not imply causality.

## Data Availability

The data supporting the findings of this study are available from the corresponding author upon reasonable request, subject to ethical and privacy considerations.

## Supplementary Materials

The following supporting information can be downloaded at: https://www.mdpi.com/article/10.3390/healthcare14152382/s1, Supplementary File S1: Author-Developed Study Measures.

## Abbreviations

The following abbreviations are used in this manuscript:

BAT-PL	Burnout Assessment Tool, Polish version
CI	Confidence interval
SD	Standard deviation
SE	Standard error
