# Supplementary material for: Death Anxiety in the Association Between Exposure-Related Burden of Patient Death and Occupational Burnout Among Healthcare Professionals: A Cross-Sectional Study
Source: Healthcare (Basel). 2026 Aug 4;14(15):2382. doi: 10.3390/healthcare14152382 (PMC13465492; doi:10.3390/healthcare14152382)
Supplement: Supplementary file 1 [file healthcare-14-02382-s001.zip › healthcare-4430245-supplementary.pdf]

Supplementary File S1

Author-Developed Study Measures

This supplementary file presents the preliminary author-developed measures used in the study: (1) the Exposure-Related Burden of Patient Death Measure and (2) the Brief Death Anxiety Measure.

1. Exposure-Related Burden of Patient Death Measure

This preliminary author-developed measure was used to assess exposure-related burden of patient death. The items refer to professional experiences related to patient death, including both exposure to such events and the subjective emotional burden associated with them. No fixed recall period was imposed; therefore, participants could refer to their broader professional experience when responding to the items.

Response format:

1 = Strongly disagree
2 = Rather disagree
3 = Neither agree nor disagree
4 = Rather agree
5 = Strongly agree

Items:

1. I witnessed the death of a patient.
2. I experienced the death of a patient with whom I had developed an emotional bond as a caregiver.
3. Following a patient's death, I experienced helplessness or feelings of guilt.
4. Following a patient's death, I found it difficult to stop thinking about the event.
5. I have access to emotional support after such events.
6. A patient's death negatively affects my motivation to work.
7. A patient's death positively affects my motivation to work.

Items 5 and 7 were reverse-coded. The total score was calculated as a mean item score. Higher scores indicate greater exposure-related burden of patient death.

2. Brief Death Anxiety Measure

This brief preliminary author-developed measure was used to assess death anxiety. The measure was developed based on theoretical concepts of death anxiety and existing instruments, particularly the Death Anxiety subscale of the Death Anxiety and Fascination Scale developed by Żemojtel-Piotrowska and Piotrowski.

Response format:

1 = Strongly disagree
2 = Rather disagree
3 = Neither agree nor disagree
4 = Rather agree
5 = Strongly agree

Items:

1. The thought of my own death causes me anxiety.

2. I am afraid of the process of dying.
3. I often worry about the death of loved ones.
4. I feel uneasy when thinking about what happens after death.
5. I try to avoid thinking about death.
6. News about a patient’s death causes tension in me.
7. I often ask myself questions about the meaning of death.
8. The thought of talking about death evokes anxiety in me.
9. The thought of accompanying someone at the time of death evokes anxiety in me.

The total score was calculated as a mean item score. Higher scores indicate greater death anxiety.
